# Toward a humanized mouse model of *Pneumocystis* pneumonia

**DOI:** 10.1172/jci.insight.139573

**Published:** 2021-01-25

**Authors:** Guixiang Dai, Alanna Wanek, Taylor Eddens, Paul Volden, Jay K. Kolls

**Affiliations:** 1 Center for Translational Research in Infection and Inflammation, Tulane School of Medicine, New Orleans, Louisiana, USA.; 2 UPMC Children’s Hospital of Pittsburgh, Pittsburgh, Pennsylvania, USA.; 3 Taconic Biosciences, Germantown, New York, USA.

**Keywords:** Infectious disease, Pulmonology, Cytokines, Fungal infections

## Abstract

*Pneumocystis* is an important opportunistic fungus that causes pneumonia in children and immunocompromised individuals. Recent genomic data show that divergence of major surface glycoproteins may confer speciation and host range selectivity. On the other hand, immune clearance between mice and humans is well correlated. Thus, we hypothesized that humanize mice may provide information about human immune responses involved in controlling *Pneumocystis* infection. CD34-engrafted huNOG-EXL mice controlled fungal burdens to a greater extent than nonengrafted mice. Moreover, engrafted mice generated fungal-specific IgM. Fungal control was associated with a transcriptional signature that was enriched for genes associated with nonopsonic recognition of trophs (*CD209*) and asci (*CLEC7A*). These same genes were downregulated in CD4-deficient mice as well as twins with bare lymphocyte syndrome with *Pneumocystis* pneumonia.

## Introduction

Animal models of *Pneumocystis* pneumonia (PCP) have been used for the discovery of new antimicrobial agents (1, 2) and vaccine testing (3, 4) as well as understanding CD4^+^ T cell immunity in the lung (5–7). It is well known in the field that speciation of the genus is associated with strict limitations in host range. That is, *Pneumocystis murina* infects mice but not rats; *Pneumocystis carinii* infects rats but not mice; and *Pneumocystis jiroveci* is the human organism. Recent genomic evidence shows that the major surface glycoproteins are the most divergent across species, and, thus, surface interactions and attachment to host cells may play a key role in limitations of host range. This is similar to nonculturable components of the gut microbiota, where segmented filamentous bacteria (SFB) from rats will not infect mice unless the strain is complemented with the attachment proteins from murine SFB (8). Interestingly, without attachment to the epithelium, rat SFB fail to elicit T cell responses in the gastrointestinal tract (8).

Despite this host range restriction, the immunology underlying organism clearance appears to be highly conserved between mice and humans (7). The epidemiology of PCP in pediatrics has changed dramatically with the development of antiretroviral therapy that has essentially eliminated HIV-associated PCP in children (7, 9). With this reduction in acquired immune deficiency, the bulk of cases of pediatric PCP in Western countries is due to primary immunodeficiency or hematological, malignancies, or treatment for connective tissue diseases (10). We recently showed a 100% concordance between human and murine loss-of-function mutation and PCP, including *Rag* and *Il21r* mutations (7). Importantly, mutations in humans that are not associated with PCP, such as *Rorc* or *Il17ra* mutations, were also not associated with failure to clear the organisms in murine models (7). In these studies, we noted that clearance was associated eosinophils (11) but also with the recruitment of macrophages that can clear *Pneumocystis* through both opsonic and nonopsonic killing of the fungus (7, 12). Based on this, we sought to determine whether newer models of humanized mice could be used to understand if this model could be used to understand fungal clearance in the lung. We thought this was relevant in not only understanding human cellular interaction, but also significant potential differences in pattern recognition between cells. For example, in humans, CD209, a mannan recognition protein, has only 1 locus with 8 isoforms mediated by splice variants, whereas, in mice, there are 7 distinct genes encoding CD209A–CD209G. To study myeloid cells, we used control or CD34-engrafted huNOG-EXL mice that are transgenic for human GM-CSF and human IL-3 (13).

## Results

### Humanized mice (huNOG-EXL mice) control Pneumocystis infection.

**Figure 1:**
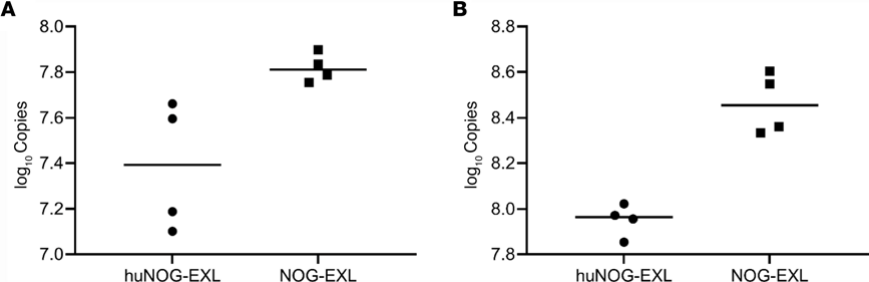
Lung fungal burden. huNOG-EXL mice engrafted with human umbilical cord blood–derived CD34+ hematopoietic stem cells and unengrafted mice were infected with 100 μl (approximately 2 × 105 asci) *P*. *murina* inoculum by oral pharyngeal aspiration. Three (**A**) and six weeks (**B**) after infection, the right middle lung lobes were removed and RNA was extracted in order to assess lung fungal burden by RT-qPCR. Analysis using a 2-tailed, unpaired *t* test indicated that there were significantly different fungal burdens between groups (4 mice each group); *P* = 0.0247 for week 3 and *P*=0.0005 for week 6. Individual values are shown with a horizontal bar indicating the mean of the group.

Both huNOG-EXL and NOG-EXL mice were infected with *P*. *murina* by oropharyngeal aspiration. Three and six weeks after infection, the right middle lung lobes were removed and RNA was extracted to assess lung fungal burden by RT-qPCR. huNOG-EXL mice showed significantly reduced fungal burden in the lungs compared with that of NOG-EXL mice at 3 weeks (Figure 1A) and 6 weeks (Figure 1B) after infection, albeit fungal burdens were still detectable in both groups of mice.

### Humanized mice generated Pneumocystis-specific IgM antibody.

**Figure 2:**
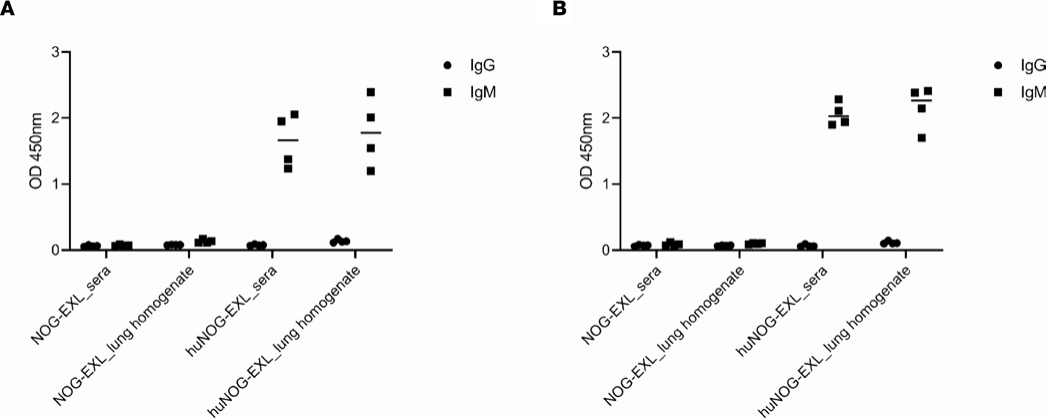
Pneumocystis antigen-specific human IgG and IgM in sera and lung homogenates. huNOG-EXL mice engrafted with human umbilical cord blood–derived CD34+ hematopoietic stem cells and unengrafted mice were infected with 100 μl (approximately 2 × 105 asci) *P*. *murina* inoculum by oral pharyngeal aspiration. Three (**A**) and six (**B**) weeks after infection, sera were collected and the right lower lung lobes were removed to assess lung homogenates. IgG and IgM in sera and lung homogenates were measured by ELISA. Sera were diluted 1:10 and 1 ml of homogenate, prepared from right lower lung lobe, was not diluted, i.e., neat, for analysis. Analysis using a 2-tailed, unpaired *t* test indicated that there were significant differences in IgG and IgM in the lung homogenates and sera between groups at both 3 weeks (**A**) and 6 week (**B**) after infection (4 mice each group); *P* < 0.0001 for all comparisons. Individual values are shown with a horizontal bar indicating the mean of the group.

Three and six weeks after mice were infected with *P*. *murina*, sera and lung homogenates were prepared and measured for *Pneumocystis*-specific human IgG and IgM. We observed detectable levels of *Pneumocystis* antigen-specific human IgM but not IgG at both 3 weeks (Figure 2A) and 6 weeks (Figure 2B) after infection. No human IgG and IgM were detected in NOG-EXL mice at 3 weeks (Figure 2A) or 6 weeks (Figure 2B) after infection, due to the fact that these mice did not have human cells.

### Human IFN-γ–, IL-4–, IL-5–, and IL-17A–secreting CD4^+^ and CD8^+^ cell responses.

**Figure 3:**
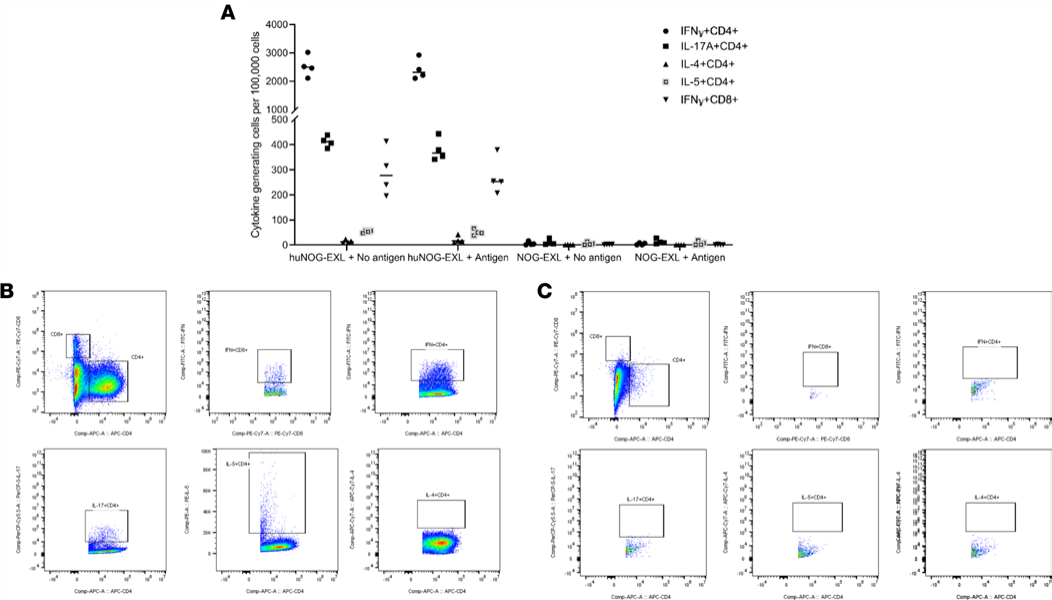
Human lymphocytes generated Pneumocystis-specific human cytokines in lung cells. huNOG-EXL mice engrafted with human umbilical cord blood–derived CD34+ hematopoietic stem cells and unengrafted mice were infected with 100 μl (approximately 2 × 105 asci) *P*. *murina* inoculum by oral pharyngeal aspiration. Six weeks after infection, single lung cells were prepared and cultured with or without *Pneumocystis* antigen for cytokine production, as measured by flow cytometry. The NOG-EXL group of 4 mice did not have human cells and, thus, no cytokine-generating human cells were detected. In vitro antigen stimulation did not have any effect on cytokine-generating cells. Analysis using a 2-tailed, unpaired *t* test (**A**) indicated that there were significant differences in cytokine-generating cells between the huNOG-EXL and NOG-EXL groups (4 mice each group); P < 0.0001 for IFN-γ+CD4+, IL-17A+CD4+, and IFN-γ+CD8+; *P* < 0.05 for IL-5+CD4+; and *P* > 0.05 for IL-4+CD4+ cells. Individual values are shown with a horizontal bar indicating the mean of the group. Representative flow cytometry gating plots are shown for huNOG-EXL (**B**) and NOG-EXL (**C**) mice.

Six weeks after mice were infected with *P*. *murina*, lung single cells were prepared and cultured with or without *Pneumocystis* antigen for cytokine production, as measured by flow cytometry. Subsets of human cytokine-expressing CD4^+^ cells and CD8^+^ cells were detected (Figure 3A). We observed production of type 1, type 2, and type 17 cytokines, with or without in vitro pneumocystis antigen stimulation, from cells of *Pneumocystis*-infected huNOG-EXL mice (Figure 3A), indicating that the cells may already be in an activated status due to exposure to *Pneumocystis* antigens in vivo. Representative gating strategies are shown in Figure 3, B and C. There were no human cytokine-expressing cells detected in unengrafted NOG-EXL mice (Figure 3, A and C).

### Human CD209 expression and lung fungal burden.

**Figure 4:**
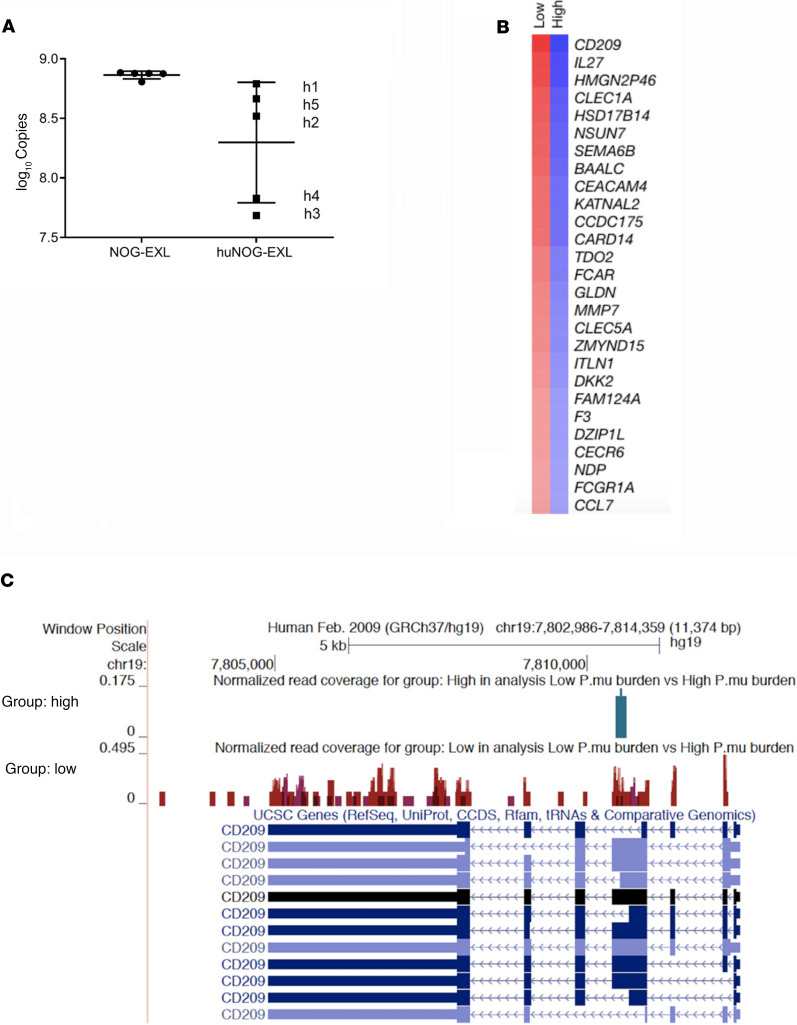
Lung RNA-Seq. A subgroup of huNOG-EXL mice appeared to clear/control *Pneumocystis murina* better at 6 weeks after infection. The whole-lung RNA-Seq h3 and h4 mouse data were compared with those for h1, h5, and h2 mice. The results indicate that lower lung fungal burden was associated with higher CD209 gene expression. h1, h5, and h2 mice had higher lung fungal burdens compared with those of h4 and h3 mice (**A**). Differently expressed genes (**B**) and human CD209 gene mapping (**C**) are shown.

Six weeks after mice were infected with *P*. *murina*, lung RNA was extracted and assayed for fungal burden. We noted that a subgroup of huNOG-EXL mice, labeled as h4 and h3 in Figure 4A, had lower *Pneumocystis* fungal burdens compared with others, labeled as h1, h5, and h2 in Figure 4A, and we hypothesized that this may due variability of engraftment. We took advantage of these differences to perform an unbiased RNA-Seq experiment comparing mice with lower fungal burdens to those with higher fungal burden 6 weeks after infection. The whole-lung RNA-Seq results indicated that lower lung fungal burden is associated with higher gene expression of *CD209* (Figure 4), which we have previously reported is pattern recognition receptor for *Pneumocystis* (7). These results were confirmed by real-time PCR (RT-PCR) (Supplemental Figure 1; supplemental material available online with this article; https://doi.org/10.1172/jci.insight.139573DS1). Pathway analysis revealed an enrichment in the following Kegg pathways: the cytokine-cytokine receptor interaction (Supplemental Figure 2) and chemokine signaling pathways (Supplemental Figure 3). We have previously reported that the murine model of *Pneumocystis* infection replicates many human mutations that confer susceptibility to infection (7). Given that the murine model is dependent on CD4^+^ T cells, we performed RNA-Seq on control mice and CD4-depleted mice at day 14, when fungal burdens are the same in the 2 groups of mice (11). Venn diagram analysis (Supplemental Figure 4) showed a core set of 55 genes that were upregulated in humanized mice and control CD4-replete mice that were associated with fungal control, including the mannan receptor, CD209; the β-glucan receptor, CLEC7A; CD14; and class II MHC (Supplemental Table 1).

Given that class II MHC is differentially expressed and also critical for *Pneumocystis* clearance, we performed RNA-Seq on BAL cells from identical twins with bare lymphocyte syndrome, due to a mutation in class II MHC, who had PCP confirmed by bronchoscopy. Compared with control BAL cells, there was a substantial decrease in genes belonging to the KEGG phagocytosis pathway, including mannose receptor, C type 1 (*MRC1*), *CSFR2*, *CD209*, and *CLEC7A* (Supplemental Figure 5).

### Pneumocystis GSC1 expression and lung Grocott’s methenamine silver staining.

**Figure 5:**
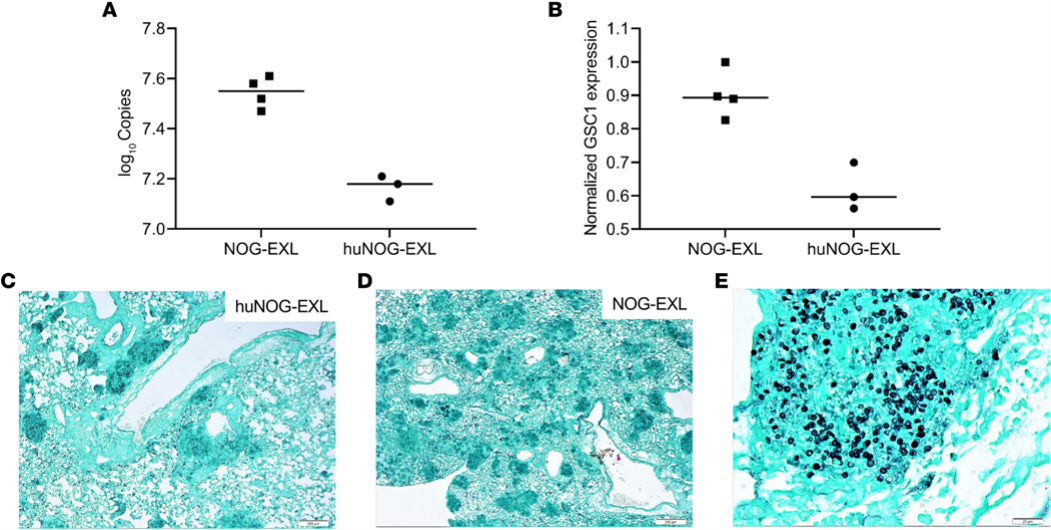
Lung fungal burden correlated with Pneumocystis GSC1 gene expression and GMS staining. huNOG-EXL mice engrafted with human umbilical cord blood–derived CD34^+^ hematopoietic stem cells and unengrafted mice were inoculated with approximately 2 × 10^5^ asci of *P*. *murina*. Eight weeks after infection, the right middle lung lobes were removed and RNA was extracted in order to assess lung fungal burden and GSC1 gene expression by RT-qPCR. The left lung lobes were taken for GMS staining. Analysis using a 2-tailed, unpaired t test indicated that there were significant differences in the fungal burden (**A**) and GSC1 gene expression (**B**) between the huNOG-EXL and NOG-EXL groups; *P* < 0.001 for fungal burden and *P* < 0.005 for GSC1 expression. Individual values are shown with a horizontal bar indicating the mean of the group. GMS staining indicated that there were more and extensive but smaller lesions in the lungs of NOG-EXL mice (**D**) compared with lungs of huNOG-EXL mice (**C**) (scale bars: 200 μm). At high magnification (original magnification, ×40), GMS-positive asci were well stained (**E**) (scale bar: 23 μm).

Eight weeks after mice were infected with *P*. *murina*, lung RNAs were extracted and assayed for lung fungal burden (Figure 5A) and *Pneumocystis* *GSC1* gene expression, which is an ascus transcript (Figure 5B). *GSC1* gene expression was significantly reduced in huNOG-EXL mice compared with that in NOG-EXL control mice(Figure 5B), and this was associated with less asci on Grocott’s methenamine silver (GMS) staining (Figure 5C). Extensive lesions with more asci were seen in NOG-EXL control mice (Figure 5D). At high magnification (original magnification, ×40), GMS-positive asci were well stained (Figure 5E).

### Mouse survival and weight recording.

All mice were weighted before and every other day after infection. Over time, both engrafted and nonengrafted mice had weight variations and lost weight, and 7 of 8 mice lost more than 20% of their initial weight at about 8 weeks after infection. According to Tulane University IACUC policy, the experiment had to be terminated at this point. Weight variations were shown as actual weight (Supplemental Figure 6A) and percentage of weight loss (Supplemental Figure 6B). There were no differences between huNOG-EXL and control NOG-EXL groups.

## Discussion

The epidemiology of pediatric PCP has changed dramatically over the last 45 years. It was initially a major complication of acute lymphoblastic leukemia and work by the Hughes lab led to the development of prophylaxis with trimethoprim-sulfamethoxazole (14). A second peak of infection occurred in the 1980; this was associated with perinatal HIV infection (15). After the development of maternal screening and the advent of retroviral therapy, PCP associated with perinatal HIV declined, and, currently, one of the major risk factors for pediatric PCP is primary immunodeficiency (10, 15). Recent genomic data from various *Pneumocystis* spp., including *P*. *carinii*, *murina*, and *jiroveci*, has shown that key aspects of the genomes such as the kinome are conserved, but it is likely that the major surface glycoproteins mediate speciation and host specificity, as these represent the most divergent proteins across species (16). In contrast to *Pneumocystis* speciation, the immune mechanisms required for clearance of primary infection appear to be very well conserved. Human mutations that confer susceptibility, such as *Il2RG*, *IL21R*, *CD40L*, and *RAG* genes, are also essential in mice (7). Based on this, we hypothesized that engrafted human cells could also mediate resistance to *P*. *murina* infection in humanized mice.

Based on previous work showing that GM-CSF (7, 17) and fungal pattern recognition receptors, such as dectin-1 (12) and CD209 (7), are important in nonopsonic phagocytosis and fungal killing, we studied huNOG-EXL mice that are transgenic for human IL-3 and GM-CSF. These mice were able to mount an IgM response but failed to mount a detectible IgG response. Engrafted mice were able to reduce the growth of *P*. *murina*, and this was associated with a *CD14*, *CLEC7A*, and *CD209* gene signature. Notably this same gene signature is associated with clearance in CD4-replete mice and humans with bare lymphocyte syndrome.

It is possible that fungal clearance was not complete in the humanized mouse model due to the lack of fungal-specific IgG, which we have shown can mediate fungal killing independent of dectin-1, via Fc receptor γ (12). The lack of class-switched immunoglobulin in the model may be due to several factors. In lung fibroblasts, *Pneumocystis* induces CXCL13 expression, which is critical for inducible bronchial-associated lymphoid tissue formation (18, 19), which may be an important site of class-switch recombination. Murine CXCL13 has only 44% amino acid identity to human CXCL13, and thus this may explain the lack of IgG in humanized mice. Additionally, both CD40L (hyper-IgM syndrome) and IL-21R mutations develop PCP, and it is possible that these important ligand-receptor relationships are impaired in this model. Thus, future studies will examine if human CXCL13 or trimeric soluble CD40L can mediate class-switch recombination in the model and the generation of fungal-specific IgG. Taken together, the humanized mouse model shows a conserved transcriptional program of pattern recognition receptor molecules that recognize β-glucan, such as CLEC7A, which recognizes the ascus, but also mannan recognition molecules, such as MRC1 and CD209, which can recognize trophic forms. Furthermore, in both CD4-deficient mice and CD4-deficient humans, the ability to generate this transcriptional program is defective. This may be due to the fact that CD4^+^ T cells can be an important source of GM-CSF (7) that not only influences alveolar macrophage function but also recruits CCR2^+^ inflammatory monocytes from the periphery. The humanized mouse model can likely be refined and improved to better understand the human immune response to PCP.

## Methods

### Mice.

Female 18- to 24-week-old huNOG-EXL mice (NOD.Cg-*Prkdc^scid^Il2rg^tm1Sug^*Tg), which originated from immunodeficient NOG mice expressing human GM-CSF and human IL-3 cytokine, engrafted with human umbilical cord blood–derived CD34^+^ hematopoietic stem cells, and nongrafted NOG-EXL mice were obtained from Taconic. Immunodeficient B10:B6-Rag2^tm1Fwa^Il2rg^tm1Wjl^ (Rag2^−/−^Il2rγ^−/−^) mice were originally obtained from Taconic and housed at the Tulane University Department of Comparative Medicine Facility. Animals were housed in a pathogen-free environment and given food and water by the DLAR ad libitum.

### Pneumocystis isolation, inoculum, and antigen preparation.

*P*. *murina* organisms were administered by oral-pharyngeal delivery to Rag2^−/−^Il2rγ^−/−^ mice, propagated for 10 to 12 weeks in vivo, and isolated from mouse lung tissue as previously described (20). Briefly, Rag2^−/−^Il2rγ^−/−^ mice with PCP were sacrificed, and the lungs were aseptically harvested and frozen in 1 ml sterile Dulbecco’s PBS at −80°C. To process the inoculum, frozen lungs were thawed, strained through a 70 μm filter, and pelleted by centrifugation (800*g*, 10 minutes, 4°C). The pellet was resuspended in 1 ml PBS. A 5 μl aliquot was diluted 1:10, heat fixed on a slide, and stained with Hema-3 modified Wright-Giemsa stain (Fisher Scientific), followed by ascus counting. *P*. *murina* asci were quantified microscopically, and the inoculum was adjusted to 2 × 10^6^ asci per ml. Mice were administered 100 μl (2 × 10^5^ asci) of the inoculum by oral-pharyngeal aspiration as previously described (20). *Pneumocystis* protein antigen was prepared by differential centrifugation of the inoculum as previously described, followed by sonication of 1 mg inoculum per ml for 5 minutes (7).

### Preparation of whole-lung cells.

Mice were infected with an inoculum of *P*. *murina* for 3 or 6 weeks. At the time of euthanasia, mice were anesthetized by inhalation of carbon dioxide. Immediately after, mice were perfused vascularly by injection of 5 ml heparinized PBS into the right ventricle. The right superior and inferior lung lobes were then harvested, minced with razor blades, and digested in 5 ml serum-free medium with 2 mg/ml collagenase for 90 minutes in a 37°C shaking incubator. The cell suspension was strained through a 70 μm filter and then washed and resuspended in complete DMEM. Red blood cells were then lysed with ammonium chloride solution, washed, resuspended in 5 ml DMEM, and counted.

### Flow cytometric analysis.

A total of 10^6^ single cells from the mouse lung were stimulated with 5 μg/ml P murina antigen for 5 to 6 hours. One hour after the start of stimulation, cells were given 1 μl/ml GolgiStop (BD Pharmingen) to block cytokine secretion. Cells were surface stained with fluorescein-conjugated antibodies for CD4 (Biolegend, 344614) and CD8 (Biolegend, 344712) for 30 minutes in PBS supplemented with 1% BSA, washed, permeated, and stained with fluorescein-conjugated antibodies for IFN-γ (Biolegend, 506504), IL-4 (Biolegend, 500834), IL-5 (Biolegend, 500904), and IL-17A (Biolegend, 512314). Cells were acquired for flow cytometry by Cytek and data were analyzed using FlowJo (Treestar).

### RNA isolation and Pneumocystis quantification by RT-PCR.

The right middle lobe of the lung was harvested in 1 ml TRIzol (Thermo Fisher Scientific) and homogenized. RNA was purified and quantified as previously described (7). Briefly, cDNA was synthesized from 1 μg whole-lung RNA via iScript reverse transcription reagents (Bio-Rad), and RT-PCR was performed using primers and probes (forward primer, 5′-TCGGACTTGGATCTTTGCTTCCCA-3′, reverse primer, 5′-CATTCCGAGAACGAACGCAATCCT-3′, probe, 5′-/56-FAM/TCATGACCC/ZEN/TTATGGAGTGGGCTACA/3IABkFQ/-3′; all from IDT) for the *P*. *murina* small-subunit (SSU) rRNA transcript and SsoAdvanced probe supermix (Bio-Rad). The threshold cycle values were converted to copy numbers by use of a premade standard of known *Pneumocystis* SSU rRNA, as previously described (7).

### Human CD209 expression by RT-PCR.

Briefly, cDNA was synthesized from 1 μg whole-lung RNA via iScript reverse transcription reagents (Bio-Rad), and RT-PCR was performed using human CD209 TaqMan Gene Expression Assays (Hs01588349_m1, Thermo Fisher Scientific). The human HPRT gene expression assay (Hs02800695_m1, Thermo Fisher Scientific) was used as reference.

### Pneumocystis GSC1 expression by RT-PCR.

cDNA synthesis was as described above. SsoAdvanced qRT-PCR universal SYBR green (Bio-Rad) was then used to quantify cDNA abundance. The forward primer was ATTATGCGCCGGAATATGG, and the reverse primer was ACTGAAGAGGACGCTGAT. The mouse HPRT gene expression assay (Mm03024075_m1, TaqMan) was used as reference.

### Bulk RNA-Seq.

RNA quantity and quality were assessed using the Qubit RNA HS Assay Kit (Invitrogen) and the Agilent RNA 6000 Nano kit with the Agilent 2100 Bioanalyzer instrument. The Illumina TruSeq Stranded mRNA sample prep kit was used for library preparation, followed by Agilent DNA 1000 kit validation with the Agilent 2100 Bioanalyzer and quantification using the Qubit 2.0 fluorometer (Invitrogen). The cDNA libraries were pooled at a final concentration 1.8 pM. Cluster generation and 75 bp paired-read single-indexed sequencing was performed on Illumina NextSeq 550. Raw reads were processed and mapped and then gene expression and nucleotide variation was evaluated by previously described methods (21). Data were deposited in the Gene Expression Omnibus (GSE159562).

### Preparation of lung homogenate.

The left lung lobe of the lung was harvested and homogenized in 1 ml solution of protease inhibitor cocktail in PBS (MilliporeSigma, SigmaFAST Protease Inhibitor Cocktail, S8830) using a power homogenizer. The homogenate was centrifuged at 10,000*g* for 5 minutes, and the supernatant was collected and stored at –80°C.

### Serum collection and P. murina antigen ELISA.

Blood was collected at the time of sacrifice by syringe from the vena cava. Coagulated blood was then centrifuged for 10 minutes at 10,000*g*. The serum supernatant was collected and stored at −80°C. Maxisorb plates were coated with 2 μg *P*. *murina* antigen in 100 μl bicarbonate-coating buffer per well overnight at 4°C. Plates were blocked with 5% blotting-grade blocker (Bio-Rad) and 1% BSA. Plates were first incubated with sample serum (1:10 dilution) or neat lung homogenate for 2 hours at room temperature with shaking and then stained with murine Ig-specific horseradish peroxidase–conjugated antibodies. Plates were then developed with tetramethylbenzidine substrate for 5 to 30 minutes, depending on the control serum, and the reaction was stopped with an equal volume of 2 N H_2_SO_4_. The optical density at 450 nm (OD_450_) was read using a Synergy H1 Hybrid reader (BioTek).

### Survival and weight recording.

All mice were weighted before *Pneumocystis* inoculation for their initial weight. After infection, all mice were weighted every other day until termination of the experiment.

### Histology.

The left lobe of lung was insufflated with 10% formalin via injection in the left main stem bronchus. The lung was then sectioned and stained with GMS stain by GNO Histology Consultants LLC. Images were taken using an Olympus DP72 microscope.

### Statistics.

GraphPad Prism (version 8.3.1) ordinary 1-way ANOVA with multiple comparisons was used to calculate *P* values. Two-group comparisons were performed with a 2-tailed, unpaired *t* test. *P* values of less than 0.05 were considered significant.

### Study approval.

All animal experiments were approved by the Tulane University IACUC (approval 4448). Human BAL and RNA-Seq studies and consent forms were approved by the University of Pittsburgh IRB (approval PRO14020408). Written informed consent was received prior to use of human samples.

## Author contributions

GD, TE, PV, and JKK designed and conducted the experiments and wrote the manuscript. AW assisted in RNA-Seq analysis and reviewed the manuscript.

## Supplementary Material

Supplemental data

Supplemental Table 1

## Version 1 01/25/2021

Electronic publication

## References

[1] Cushion MT (2010). Echinocandin treatment of *Pneumocystis* pneumonia in rodent models depletes cysts leaving trophic burdens that cannot transmit the infection. PLoS One.

[2] O’Leary TJ (1995). Use of semiquantitative PCR to assess onset and treatment of *Pneumocystis carinii* infection in rat model. J Clin Microbiol.

[3] Wells J (2006). Active immunization against *Pneumocystis*
*carinii* with a recombinant P. carinii antigen. Infect Immun.

[4] Tesini BL (2017). Immunization with *Pneumocystis* cross-reactive antigen 1 (Pca1) protects mice against *Pneumocystis* pneumonia and generates antibody to *Pneumocystis*
*jirovecii*. Infect Immun.

[5] Kelly MN (2013). Memory CD4+ T cells are required for optimal NK cell effector functions against the opportunistic fungal pathogen *Pneumocystis*
*murina*. J Immunol.

[6] Shellito JE (2000). Murine CD4+ T lymphocyte subsets and host defense against *Pneumocystis*
*carinii*. J Infect Dis.

[7] Elsegeiny W (2018). Murine models of *Pneumocystis* infection recapitulate human primary immune disorders. JCI Insight.

[8] Atarashi K (2015). Th17 Cell induction by adhesion of microbes to intestinal epithelial cells. Cell.

[9] Zakrzewska M (2019). *Pneumocystis* pneumonia: still a serious disease in children. Dev Period Med.

[10] Ling C (2018). *Pneumocystis* pneumonia in non-HIV children: a 10-year retrospective study. Clin Respir J.

[11] Eddens T (2015). Eosinophils contribute to early clearance of *Pneumocystis*
*murina* infection. J Immunol.

[12] Steele C (2003). Alveolar macrophage-mediated killing of *Pneumocystis*
*carinii* f. sp. muris involves molecular recognition by the dectin-1 beta-glucan receptor. J Exp Med.

[13] Perdomo-Celis F (2019). HIV replication in humanized IL-3/GM-CSF-transgenic NOG mice. Pathogens.

[14] Hughes WT (1977). *Pneumocystis**carinii* pneumonia. N Engl J Med.

[15] Garcia-Moreno J (2020). *Pneumocystis**jirovecii* pneumonia in children. A retrospective study in a single center over three decades. Enferm Infecc Microbiol Clin.

[16] Eddens T (2019). Transcriptomic and proteomic approaches to finding novel diagnostic and immunogenic candidates in *Pneumocystis*. mSphere.

[17] Mandujano JF (1995). Granulocyte-macrophage colony stimulating factor and *Pneumocystis*
*carinii* pneumonia in mice. Am J Respir Crit Care Med.

[18] Eddens T (2017). *Pneumocystis*-driven inducible bronchus-associated lymphoid tissue formation requires Th2 and Th17 immunity. Cell Rep.

[19] Rangel-Moreno J (2011). The development of inducible bronchus-associated lymphoid tissue depends on IL-17. Nat Immunol.

[20] Zheng M (2014). Novel *Pneumocystis* antigen discovery using fungal surface proteomics. Infect Immun.

[21] Butler A (2018). Integrating single-cell transcriptomic data across different conditions, technologies, and species. Nat Biotechnol.

