# Supplementary material for: Toward a humanized mouse model of Pneumocystis pneumonia
Source: JCI Insight. 2021 Jan 25;6(2):e139573. doi: 10.1172/jci.insight.139573 (PMC7934868; doi:10.1172/jci.insight.139573)
Supplement: Supplemental data [file jciinsight-6-139573-s218.pdf]

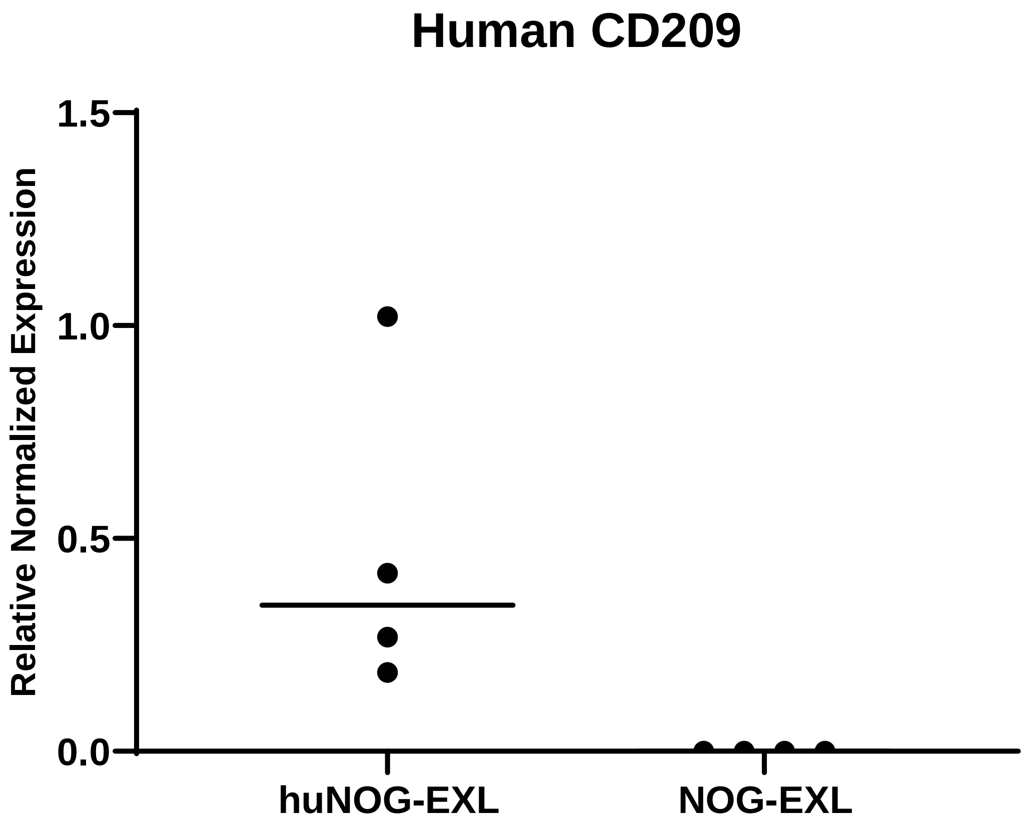

Figure S1. Human CD209 expression. huNOG-EXL engrafted with human umbilical cord blood-derived CD34+ hematopoietic stem cells (HSCs) and un-engrafted mice were infected with 100 microliter (approximately $2x10^5$ asci) of *P. murina* inoculum by oral pharyngeal aspiration. Six weeks post-infection, the lung RNA were extracted and tested for human CD209 gene expression by qRT-PCR. CD209 is a C-type lectin receptor present on the surface of both dendritic cells and macrophages, which is presumed to be the key molecule for cell activation by pneumocystis mannans. Analysis using a 2-tailed, unpaired t test indicated that there were significantly different in the human CD209 gene expression between groups, 4 mice each group, $P < 0.03$. In the figure, individual values are shown with a horizontal indicating the mean of the group.

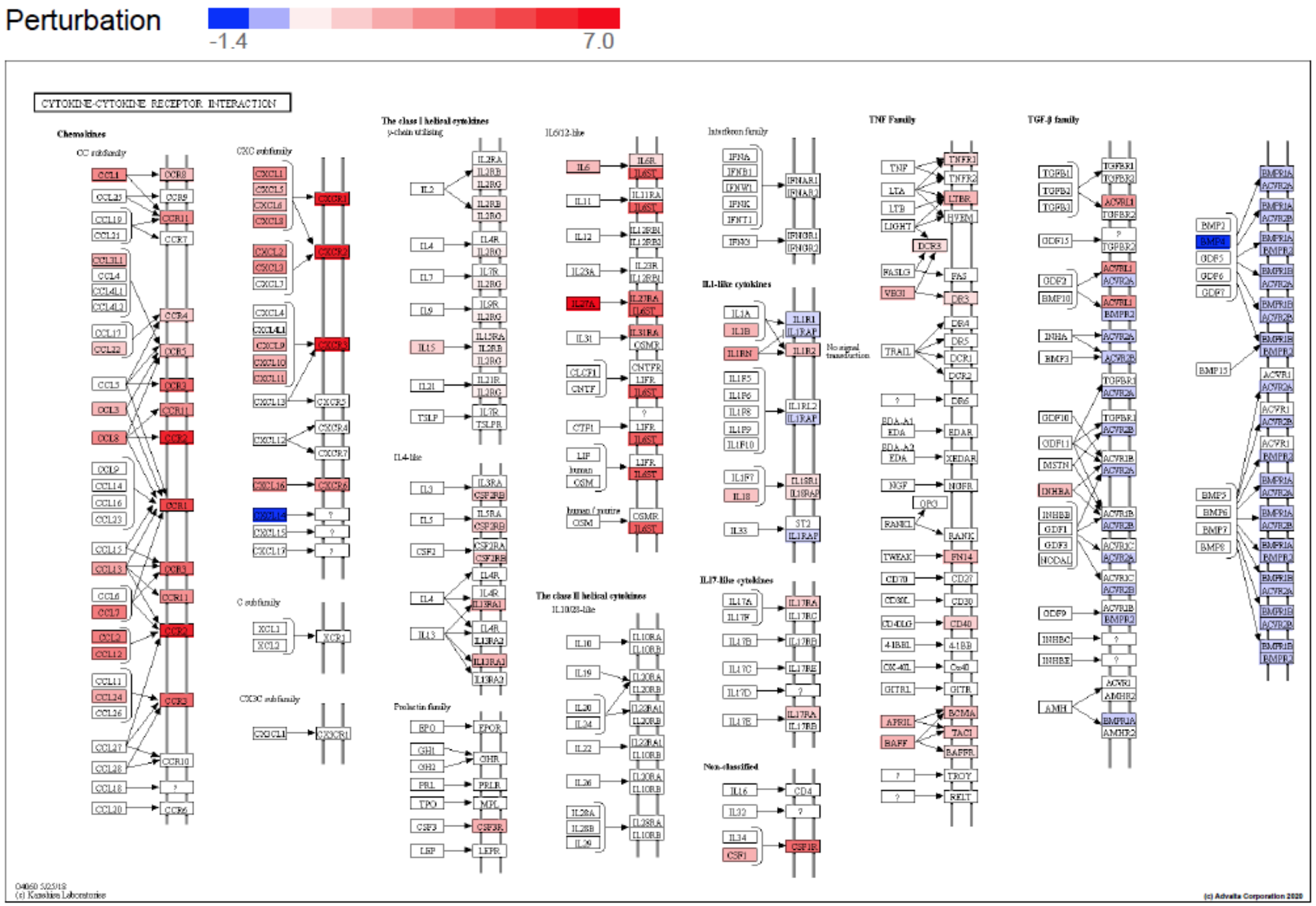

Figure S2. Differentially expressed genes in the Cytokine-cytokine receptor interaction (KEGG: 04060) in humanized mice.

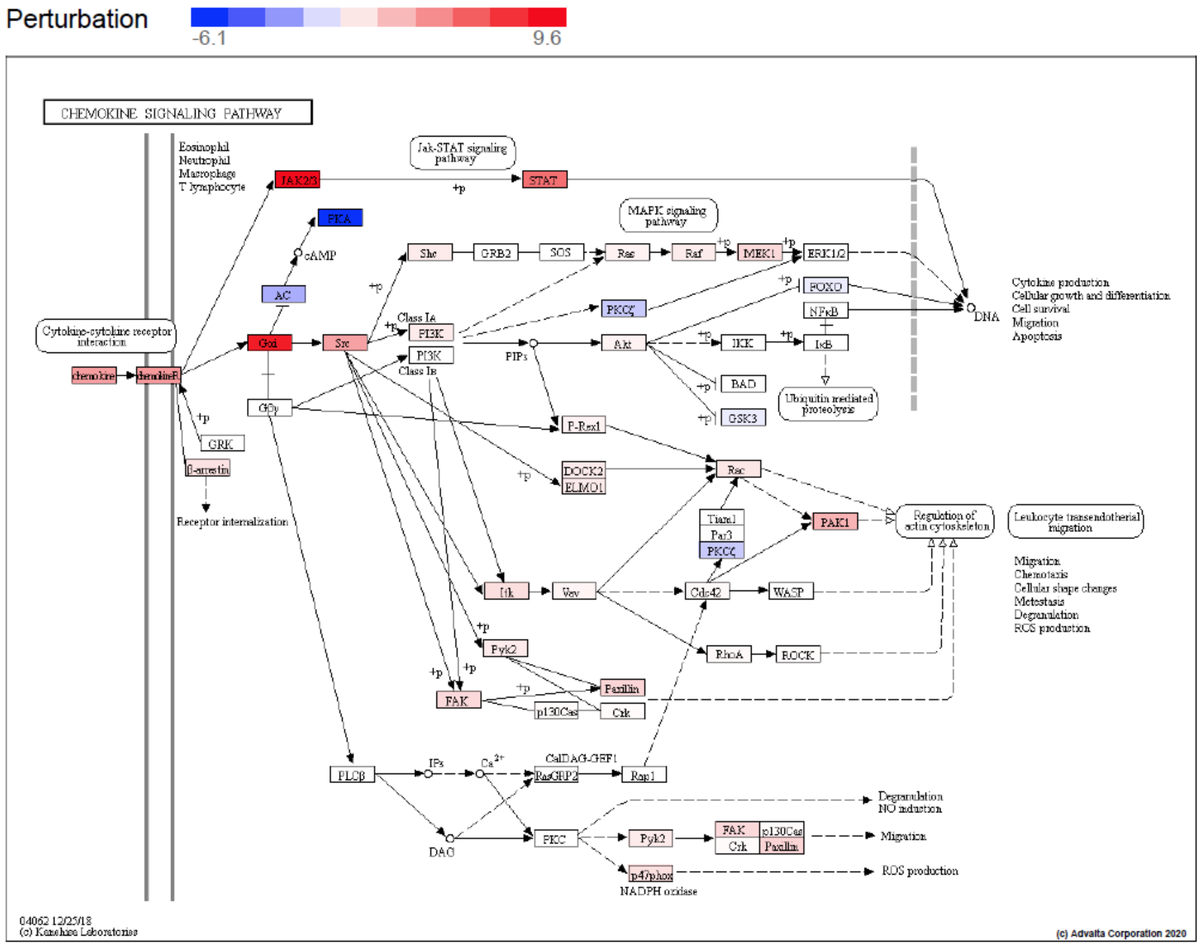

Figure S3. Differentially expressed genes in the Chemokine signaling pathway (KEGG: 04062) in humanized mice

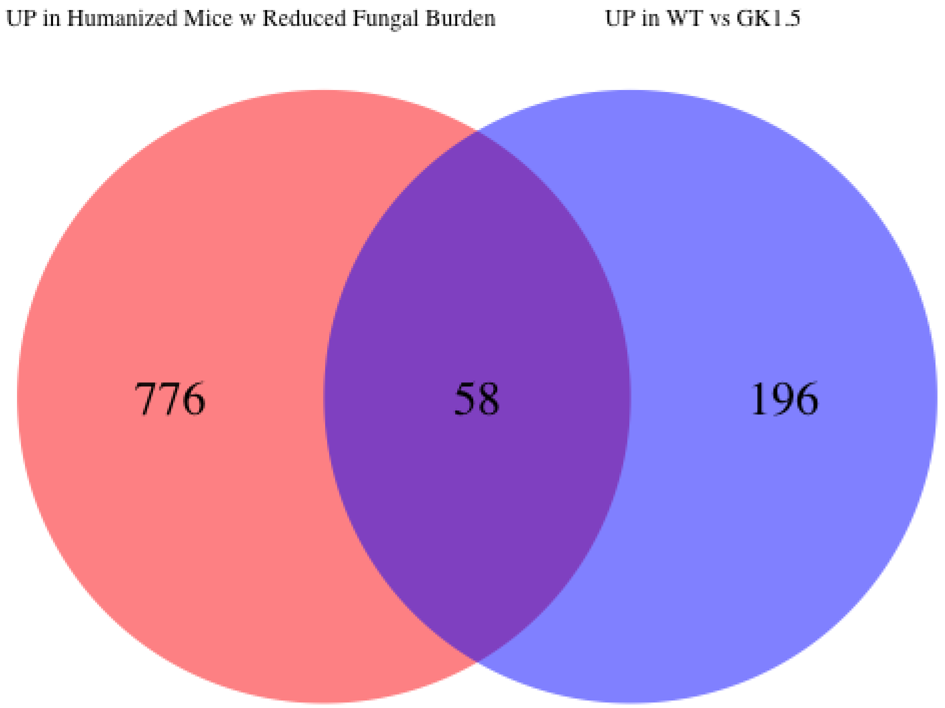

Figure S4. VENN diagram comparisons of upregulated genes in whole lung tissue in CD4 replete mice and humanized mice that control Pneumocystis infection.

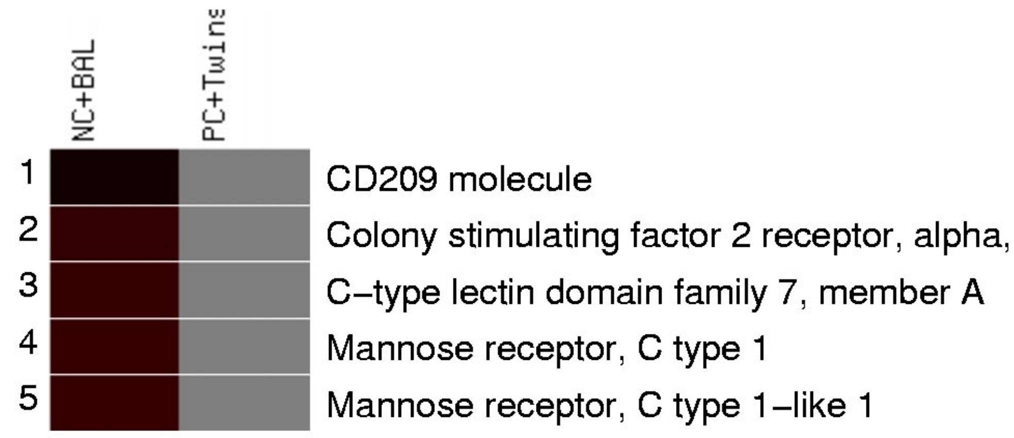

Figure S5. Differential gene expression in BAL cells from identical twins with Bare lymphocyte syndrome with PCP compared to control BAL from research bronchoscopy samples. The gray in the heat map is equivalent to less than 10 reads mapping to that gene.

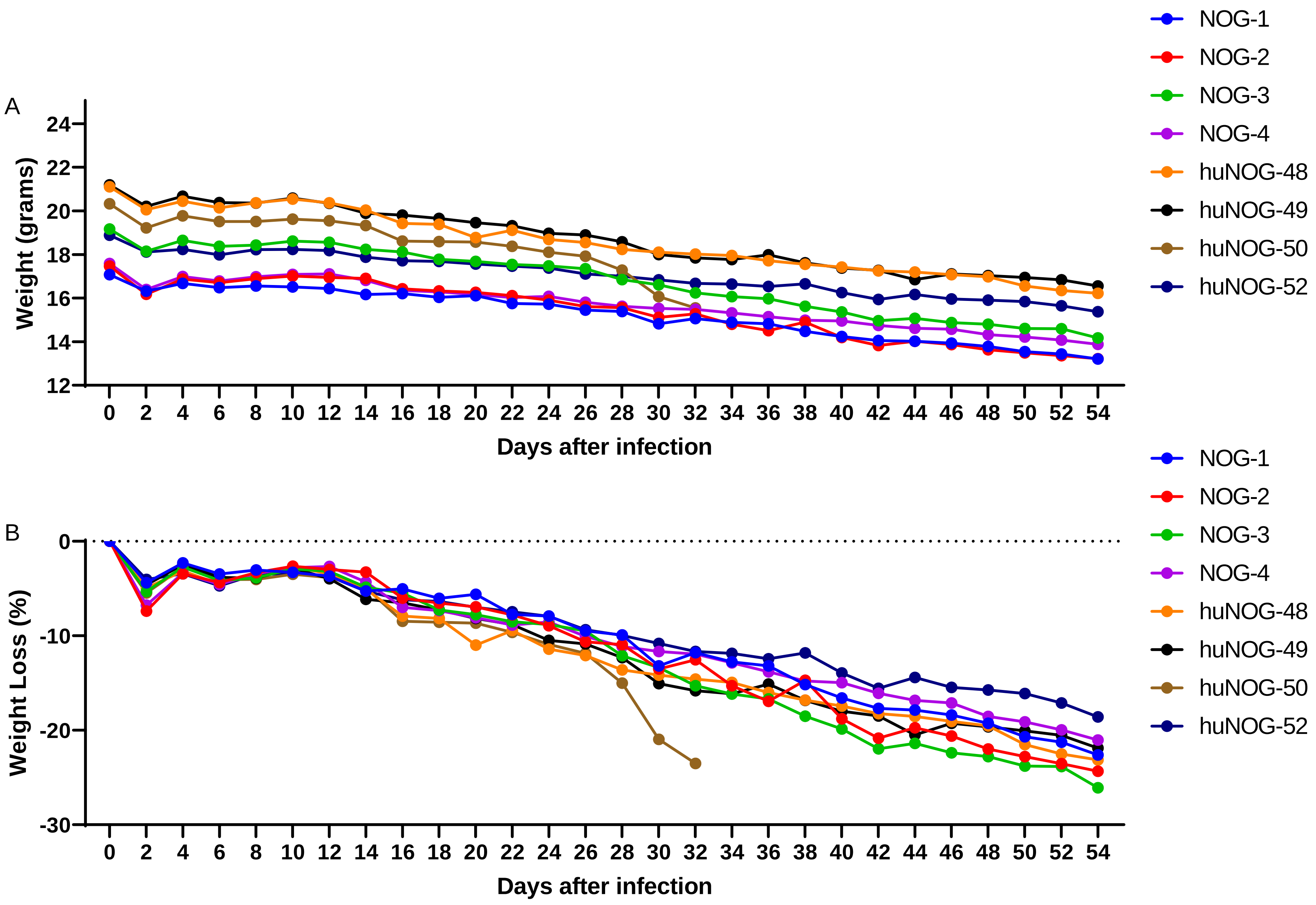

Figure S6. Weight recording. huNOG-EXL mice engrafted with human umbilical cord blood-derived CD34+ hematopoietic stem cells (HSCs) and un-engrafted mice were inoculated with approximately $2x10^5$ asci of *P. murina* inoculum. All mice were weighed every other day until weight loss exceeded 20% for 7 of 8 mice at 8 weeks after infection. huNOG-50 died at day 32 post-infection.
