# Supplementary material for: Toward a humanized mouse model of Pneumocystis pneumonia
Source: JCI Insight. 2021 Jan 25;6(2):e139573. doi: 10.1172/jci.insight.139573 (PMC7934868; doi:10.1172/jci.insight.139573)
Supplement: Supplemental Table 1 [file jciinsight-6-139573-s219.pdf]

Supplemental Table I

UPinWTvG	UPinHuMlowFB	overlap
FAR1	CD209	PLEKHO2
B2M	IL27	GCLC
CALR	HMGN2P46	ME1
XPO1	CLEC1A	NEK6
PDIA3	HSD17B14	TMEM106A
SERPINH1	NSUN7	KCNJ15
PLEKHO2	SEMA6B	SH3PXD2B
HSPD1	BAALC	RHOU
SYVN1	CEACAM4	TGFBI
DNAJB9	KATNAL2	CTSZ
SLC25A19	CCDC175	SULF2
MHCII	CARD14	P2RY6
MDN1	TDO2	CD74
GCLC	FCAR	HLA-DQB1
PTPN2	GLDN	CD14
CHORDC1	MMP7	CSF2RB
LOX	CLEC5A	MRC1
STIP1	ZMYND15	ITGAX
BPIFB1	ITLN1	SLAMF7
ME1	DKK2	CD84
PDIA6	FAM124A	FCGR2A
HYOU1	F3	CLEC10A
NLRC5	DZIP1L	IFI30
NEK6	CECR6	CLEC4A
SLC39A14	NDP	CLEC7A
TMEM106,	FCGR1A	TFEC
KCNJ15	CCL7	C1QC
SH3PXD2B	CRABP2	C1QA
RHOU	PIPOX	FOLR2
LRP2	CCL2	CH25H
MVD	VSIG4	C1QB
TGFBI	LINC01010	SIGLEC1
NFKB2	DPRXP4	CTSS
C5	FAM20A	CCR1
HSP90B1	ANKRD29	SLAMF8
ALDH18A1	OCSTAMP	MS4A7
EDEM1	TGM2	HK3
CTSZ	TM4SF19	CXCL10
C6	TRPV4	CTSK
AACS	CCL8	MSR1
PPA1	CCL1	STEAP4
PIK3R5	KCNE1	CXCL9
RASAL3	SLC9A7P1	MS4A6A
DOCK11	CHI3L1	TIMP1
BHLHE40	LOC731424	CD209

HCLS1	DCSTAMP	CCL7
FASN	MSR1	PDCD1LG2
PDIA4	IL31RA	CCL2
ITK	CXCL3	CXCL2
CRELD2	TREM2	C15orf48
SFTPD	MGST1	GPR84
BCL3	METTL7B	CXCL5
SULF2	TMEM86A	OCSTAMP
CYP51A1	A2M	SERPINA1
CREB3L1	AQP9	MMP12
DUSP2	NUPR1	CCL8
ADAM8	FHAD1	CCL24
P2RY6	YPEL4	FBP1
KCNAB2	FBP1	NA
NFKBIE	LOC100506585	NA
FSCN1	CXCL16	NA
MANF	RAB13	NA
SLC5A3	LOC391322	NA
CTSC	IL8	NA
COTL1	MS4A4A	NA
HSPA5	SERPING1	NA
MUC5B	PLA2G4C	NA
CD74	CA12	NA
HLA-DQB1	GBP1P1	NA
SLC7A2	C11orf45	NA
FABP5	ACVRL1	NA
CIITA	SPP1	NA
RAB3IL1	TLN2	NA
HSPE1	NDRG2	NA
SCD	C15orf48	NA
ITIH4	KCNJ15	NA
SERPINA3	MEIS3P1	NA
LAG3	IL1RN	NA
FOXM1	HNMT	NA
CD14	CYP27B1	NA
RRM2	CDCP1	NA
ABCD2	FOLR2	NA
FCRL2	ECM1	NA
PDE3B	ADAMDEC1	NA
MTHFD2	CD300LF	NA
CSF2RB	CFH	NA
UHRF1	HCAR2	NA
COL15A1	CD163	NA
MRC1	FAM198B	NA
CCDC80	CMKLR1	NA
IL21R	C1QB	NA
PRUNE2	PRAM1	NA

ITGAX	HCAR3	NA
SLAMF7	SIGLEC1	NA
F7	AK4	NA
CD84	CTSL	NA
REG3G	CLEC4D	NA
FCGR2A	SUCNR1	NA
FIGNL1	NRIP3	NA
CLEC10A	RSAD2	NA
IFI30	MMP12	NA
RAD51	HBEGF	NA
P2RY10	NANOS1	NA
SH2B2	LILRB5	NA
MAFB	NLRP2	NA
CSF2RB	LILRA2	NA
CD83	CXCL1	NA
CLEC4A	FGD5	NA
CFB	FAM213A	NA
CCR5	IDO1	NA
F13A1	CYP1B1	NA
ACP5	PPIC	NA
RNASE2	PLAUR	NA
DOK2	MMP9	NA
CLEC7A	FCER1G	NA
TFEC	SLC47A1	NA
KRT5	TRPM4	NA
C1QC	SLC11A1	NA
SMPD3	PTGR1	NA
C1QA	ZNF223	NA
FOLR2	LHFPL2	NA
CH25H	UACA	NA
C1QB	ABCC3	NA
SIGLEC1	PROS1	NA
IL33	MFSD7	NA
IFI16	CLEC4E	NA
PIGR	KCNMA1	NA
CTSS	MRC1	NA
CCR1	LILRB4	NA
SLAMF8	GRIN3A	NA
C3	TNFAIP6	NA
IGF1	TMTC1	NA
CLDN22	PILRA	NA
CFP	OLR1	NA
FCGR2B	AVPI1	NA
GAPT	TLR8	NA
KCNN4	PLXDC2	NA
MS4A7	HK3	NA
CD5	PSTPIP2	NA

HK3	ATF3	NA
SERPINA3	CCL13	NA
GP2	CD33	NA
TACR1	CD14	NA
TNIP3	CHIT1	NA
CXCL10	IFIT3	NA
CCL15	ANKRD22	NA
CTSK	SLC16A5	NA
HTR7	GPR84	NA
SDF2L1	ITGB5	NA
CLEC4A	IFI27	NA
MSR1	B3GNT5	NA
STEAP4	SERPINE1	NA
CXCL9	EPB41L3	NA
CD4	SPHK1	NA
RIMKLA	C1S	NA
DHRS9	CD68	NA
RGS16	ZNF618	NA
BATF3	TLR2	NA
MS4A6A	CDS1	NA
TIMP1	MRAS	NA
GATM	RAB20	NA
PTGER3	PADI2	NA
NNAT	RNASE1	NA
F10	SNX10	NA
ADRA2A	CXCL2	NA
CCL17	C1QC	NA
ALOX15	EPHB2	NA
CD209	APOE	NA
GJB5	MFAP5	NA
AOC3	SDC3	NA
ALB	EMP1	NA
SLC5A1	CCL24	NA
CHL1	C3AR1	NA
MAB21L3	SLAMF8	NA
GDF5	SLC28A3	NA
RASAL1	PVRL2	NA
THRSP	NR1H3	NA
UBD	PTAFR	NA
NXPE1	BST1	NA
GPR55	CH25H	NA
TUBB3	NCEH1	NA
ANOS1	CXCL9	NA
CYP2R1	C5AR1	NA
MZB1	STARD8	NA
STAC2	GPNMB	NA
CFD	CXCL11	NA

LCN2	PLA2G7	NA
DNASE1L3	SERINC2	NA
MPZ	SLC22A15	NA
CCL7	CLEC7A	NA
ACTA1	LILRA3	NA
KRT17	FN1	NA
CD5L	CA2	NA
RETN	FCGR1B	NA
PDCD1LG2	FMN1	NA
PNPLA3	SORBS3	NA
FABP4	SLC31A1	NA
ORM1	KCNMB1	NA
CCL2	TMEM51	NA
LIPC	TYROBP	NA
CCL23	TMEM144	NA
CXCL2	MARCO	NA
PLIN1	LILRB3	NA
ANKRD55	LAIR1	NA
ALOX15	HSD11B1	NA
HLA-A	PDGFC	NA
PLA2G2D	FCGR3A	NA
NFASC	GP1BA	NA
C15orf48	CD274	NA
CA3	MCOLN3	NA
GPR84	CLEC6A	NA
ADIPOQ	SIRPA	NA
CES1	TMEM53	NA
CCL11	MMP19	NA
SYT13	C1QA	NA
GBP2	CSF3R	NA
FCN1	SLC12A8	NA
IL10	BACE1	NA
FXYD4	IGSF6	NA
KLB	ZMIZ1-AS1	NA
PCK1	CSF1	NA
CXCL13	NFAM1	NA
LGALS7	ST3GAL6	NA
TNFRSF17	RTN1	NA
LY6H	TLR4	NA
CAPN9	SLC15A3	NA
MRGPRG	SOD2	NA
BTBD17	ASAP2	NA
PRG2	HSD3B7	NA
CCL2	LPCAT2	NA
COL6A5	SIGLEC9	NA
DERL3	RBMS2	NA
MUC5AC	ALDH3B1	NA

CCR3	CXCR2P1	NA
SAA1	TNFSF13B	NA
CXCL5	TTC23	NA
LEP	SORT1	NA
SLC26A4	ZNF804A	NA
CD209	COL8A2	NA
SAA4	SERPINA1	NA
OCSTAMP	CXCL10	NA
OOSP1	ITGAM	NA
FGF23	FPR3	NA
EBF3	SLCO2B1	NA
RETNLB	PLBD1	NA
SERPINA1	PLAU	NA
MMP12	IL1B	NA
ARG1	RHOBTB1	NA
CCL8	SLC1A3	NA
CCL24	ALDH1A1	NA
KRT76	SLC40A1	NA
FBP1	CYP2S1	NA
AWAT1	PPARGC1B	NA
GLYCAM1	SLC1A2	NA
NA	undefined	NA
NA	CD276	NA
NA	NCF2	NA
NA	STEAP3	NA
NA	LTBR	NA
NA	SLC7A7	NA
NA	FTH1	NA
NA	ARRDC4	NA
NA	CCL3	NA
NA	IL13RA1	NA
NA	FPR2	NA
NA	ADAMTSL4	NA
NA	AHNAK2	NA
NA	CSF1R	NA
NA	ACO1	NA
NA	TNFSF13	NA
NA	LOC100505702	NA
NA	ZNF19	NA
NA	RENBP	NA
NA	CPM	NA
NA	PLTP	NA
NA	PAPLN	NA
NA	TREM1	NA
NA	SCARF1	NA
NA	SLC8A1	NA
NA	LILRA6	NA

NA	GLUL	NA
NA	CYP27A1	NA
NA	RILP	NA
NA	SMIM3	NA
NA	DYSF	NA
NA	FCGRT	NA
NA	RAB39A	NA
NA	SRXN1	NA
NA	SPINT1	NA
NA	KCNJ2	NA
NA	IL4I1	NA
NA	SLC31A2	NA
NA	LRRC25	NA
NA	SEMA3C	NA
NA	C1orf162	NA
NA	STON2	NA
NA	ADAP2	NA
NA	TBC1D2	NA
NA	SCPEP1	NA
NA	FCGR2A	NA
NA	SLC39A8	NA
NA	ASAH1	NA
NA	PDCD1LG2	NA
NA	TGFA	NA
NA	IL6	NA
NA	CARD9	NA
NA	CCDC170	NA
NA	PAPSS2	NA
NA	ENG	NA
NA	SCARB2	NA
NA	METTL21B	NA
NA	ANPEP	NA
NA	LILRA5	NA
NA	CTSB	NA
NA	TNFSF15	NA
NA	CSPG4	NA
NA	IFIT2	NA
NA	GAS2L1	NA
NA	TMEM176B	NA
NA	TNFRSF12A	NA
NA	GALNT12	NA
NA	FARP1	NA
NA	APOL4	NA
NA	KCNK13	NA
NA	HK2	NA
NA	RRAGD	NA
NA	KIFC3	NA

NA	CLEC12A	NA
NA	PIWIL4	NA
NA	CREG1	NA
NA	TNS3	NA
NA	THBD	NA
NA	TFCP2L1	NA
NA	GNS	NA
NA	S100A9	NA
NA	LPL	NA
NA	CXCL5	NA
NA	FTL	NA
NA	NRP1	NA
NA	KIAA1522	NA
NA	LIPA	NA
NA	CTSD	NA
NA	P2RY13	NA
NA	PLEKHA7	NA
NA	SECTM1	NA
NA	CYBB	NA
NA	FMNL2	NA
NA	CSTB	NA
NA	DSC2	NA
NA	DAGLA	NA
NA	AGRN	NA
NA	OLFML2B	NA
NA	GGTA1P	NA
NA	SLC37A2	NA
NA	EPHX1	NA
NA	ADAM9	NA
NA	ELOVL7	NA
NA	SLC29A3	NA
NA	BATF2	NA
NA	ETS2	NA
NA	CDC42EP1	NA
NA	INHBA	NA
NA	GSN	NA
NA	GGT5	NA
NA	PFKFB4	NA
NA	RNF144B	NA
NA	CLEC10A	NA
NA	SIGLEC7	NA
NA	LYZ	NA
NA	ALAS1	NA
NA	FABP3	NA
NA	FAM129B	NA
NA	PDLIM4	NA
NA	CBS	NA

NA	IFI6	NA
NA	FAM26F	NA
NA	MYOF	NA
NA	PCSK5	NA
NA	DRAM1	NA
NA	TCN2	NA
NA	EMR2	NA
NA	ITGAX	NA
NA	IL18	NA
NA	ALDH1A2	NA
NA	ME1	NA
NA	ICAM1	NA
NA	EMR4P	NA
NA	TGFBI	NA
NA	GK	NA
NA	P2RY6	NA
NA	SLC12A7	NA
NA	VPS9D1	NA
NA	GAS2L3	NA
NA	CTSS	NA
NA	TSPAN4	NA
NA	SH3PXD2B	NA
NA	EMILIN2	NA
NA	MOB3B	NA
NA	MLPH	NA
NA	B3GNT7	NA
NA	GBP5	NA
NA	MS4A14	NA
NA	BAIAP2-AS1	NA
NA	NLRP3	NA
NA	ADAMTS14	NA
NA	SPIRE1	NA
NA	HEBP1	NA
NA	RHOU	NA
NA	RASGEF1B	NA
NA	ENPP4	NA
NA	IL18BP	NA
NA	ASPH	NA
NA	PGD	NA
NA	SNTB1	NA
NA	GM2A	NA
NA	DMXL2	NA
NA	IRAK3	NA
NA	LYRM9	NA
NA	MITF	NA
NA	GRN	NA
NA	KYNU	NA

NA	P2RX7	NA
NA	FAM20C	NA
NA	S100A8	NA
NA	ALPK1	NA
NA	FNIP2	NA
NA	CMPK2	NA
NA	SIPA1L2	NA
NA	CCRL2	NA
NA	PARP3	NA
NA	GLB1L	NA
NA	CTSZ	NA
NA	SLAMF7	NA
NA	MB21D2	NA
NA	QPCT	NA
NA	ABCG1	NA
NA	EPDR1	NA
NA	ZNF438	NA
NA	CCR1	NA
NA	TNFAIP2	NA
NA	SLC27A3	NA
NA	SLC16A3	NA
NA	MS4A7	NA
NA	KLHDC7B	NA
NA	CCND1	NA
NA	LILRB2	NA
NA	DOCK4	NA
NA	TNS1	NA
NA	ACP2	NA
NA	DDIT4L	NA
NA	LOC728431	NA
NA	TM6SF1	NA
NA	ACSL1	NA
NA	PLA2G15	NA
NA	ZNF365	NA
NA	GMPR	NA
NA	VASH1	NA
NA	KCNE3	NA
NA	CD1B	NA
NA	SRC	NA
NA	PLXND1	NA
NA	ALDH2	NA
NA	SDC2	NA
NA	OSCAR	NA
NA	PSAP	NA
NA	SEPN1	NA
NA	ANK2	NA
NA	RASSF4	NA

NA	GPC4	NA
NA	DOCK1	NA
NA	RBM47	NA
NA	PALLD	NA
NA	TFEC	NA
NA	NPL	NA
NA	ATF5	NA
NA	SCIMP	NA
NA	NLRC4	NA
NA	LGALS2	NA
NA	ABL2	NA
NA	TMEM176A	NA
NA	MATK	NA
NA	MAFF	NA
NA	ACOT4	NA
NA	CSF2RB	NA
NA	MNDA	NA
NA	IFI44L	NA
NA	NBPF1	NA
NA	HMOX1	NA
NA	ATP6V1B2	NA
NA	VPS37C	NA
NA	BRI3	NA
NA	CDKN1A	NA
NA	FLVCR2	NA
NA	SOWAHC	NA
NA	GBP1	NA
NA	NPC2	NA
NA	NEK6	NA
NA	KIF13A	NA
NA	ST14	NA
NA	IFIT1	NA
NA	NCKAP5L	NA
NA	S100B	NA
NA	10-Sep	NA
NA	PDK4	NA
NA	MFSD12	NA
NA	GAA	NA
NA	NACC2	NA
NA	HEXB	NA
NA	CCL22	NA
NA	RHOBTB3	NA
NA	ARHGEF11	NA
NA	AP5B1	NA
NA	DOCK5	NA
NA	CLEC4A	NA
NA	TLR1	NA

NA	AGPAT9	NA
NA	ITGA5	NA
NA	WARS	NA
NA	ATP6AP1	NA
NA	C1orf54	NA
NA	SLC39A11	NA
NA	LRP1	NA
NA	MSRA	NA
NA	FUCA1	NA
NA	CLCN7	NA
NA	TNKS1BP1	NA
NA	TTYH3	NA
NA	LEPROT	NA
NA	IFI30	NA
NA	PDE4A	NA
NA	LRP12	NA
NA	IL17RA	NA
NA	SLC38A7	NA
NA	APOL1	NA
NA	BCL2A1	NA
NA	EGR2	NA
NA	APOBR	NA
NA	KCTD12	NA
NA	RHOC	NA
NA	CD40	NA
NA	IL15	NA
NA	DAB2	NA
NA	CLN8	NA
NA	QSOX1	NA
NA	ZFYVE16	NA
NA	PLA2G4A	NA
NA	CORO1C	NA
NA	SDC4	NA
NA	PLD3	NA
NA	RMDN3	NA
NA	FGR	NA
NA	SPG20	NA
NA	DIRC2	NA
NA	STOM	NA
NA	RXRA	NA
NA	ITPRIPL2	NA
NA	SDCBP	NA
NA	UPP1	NA
NA	OSBPL1A	NA
NA	STAC	NA
NA	PLXNB2	NA
NA	SLC43A2	NA

NA	STEAP4	NA
NA	SGMS2	NA
NA	IGF2R	NA
NA	MVB12B	NA
NA	DNASE2	NA
NA	AXL	NA
NA	CAPG	NA
NA	FCHO2	NA
NA	SLC2A6	NA
NA	SLC38A6	NA
NA	MX1	NA
NA	FGD4	NA
NA	CST3	NA
NA	NCF1	NA
NA	MMP14	NA
NA	RASSF8	NA
NA	PLEK	NA
NA	SLC36A1	NA
NA	SYNC	NA
NA	LAP3	NA
NA	ABCA1	NA
NA	P2RX4	NA
NA	RIN2	NA
NA	P4HA2	NA
NA	ALOX5	NA
NA	RIPK2	NA
NA	AMPD3	NA
NA	SPECC1	NA
NA	RAB31	NA
NA	PLXNA1	NA
NA	FGL2	NA
NA	MKNK1	NA
NA	LGALS9	NA
NA	GPD2	NA
NA	PTPRE	NA
NA	SLC26A11	NA
NA	PPARD	NA
NA	DDX60L	NA
NA	RNF135	NA
NA	IFI44	NA
NA	APLP2	NA
NA	SMPDL3A	NA
NA	ARRB1	NA
NA	G6PD	NA
NA	PPT1	NA
NA	SULF2	NA
NA	FGD6	NA

NA	ARL11	NA
NA	GAB2	NA
NA	CTSK	NA
NA	MTHFR	NA
NA	C1RL	NA
NA	ARHGEF10L	NA
NA	VCAM1	NA
NA	FUT4	NA
NA	PRRG4	NA
NA	RHOQ	NA
NA	ATP6V0C	NA
NA	DPYD	NA
NA	LILRB1	NA
NA	SCRN1	NA
NA	GSAP	NA
NA	ABHD2	NA
NA	PTPRO	NA
NA	SQRDL	NA
NA	FAM105A	NA
NA	RAB32	NA
NA	S100A11	NA
NA	FUCA2	NA
NA	PSD3	NA
NA	SFXN3	NA
NA	TMEM106A	NA
NA	ATP6V1A	NA
NA	GBA	NA
NA	PDXK	NA
NA	ZC3H12A	NA
NA	LPAR1	NA
NA	HLA-DQA1	NA
NA	PLD1	NA
NA	CTSO	NA
NA	CTNS	NA
NA	NPC1	NA
NA	HLA-DRB3	NA
NA	OAS3	NA
NA	SH3BP2	NA
NA	LITAF	NA
NA	CD81	NA
NA	SLC8B1	NA
NA	HCK	NA
NA	P2RY14	NA
NA	C20orf194	NA
NA	PLBD2	NA
NA	LAMP1	NA
NA	NAIP	NA

NA	CALML4	NA
NA	ATP11A	NA
NA	B4GALT5	NA
NA	MAPK13	NA
NA	DSE	NA
NA	PLEKHG3	NA
NA	TYMP	NA
NA	ACE	NA
NA	ATP6V1F	NA
NA	SLC11A2	NA
NA	NRIP1	NA
NA	IRAK2	NA
NA	SLC37A1	NA
NA	LACC1	NA
NA	TXN	NA
NA	NHLRC3	NA
NA	FKBP5	NA
NA	STX11	NA
NA	LGALS3BP	NA
NA	PIK3AP1	NA
NA	ARHGAP31	NA
NA	OGFRL1	NA
NA	RALB	NA
NA	ARSB	NA
NA	LIMK2	NA
NA	TSPO	NA
NA	DUSP3	NA
NA	DAPK1	NA
NA	TNFRSF1A	NA
NA	KIAA0930	NA
NA	TOM1	NA
NA	TPK1	NA
NA	ADAM15	NA
NA	FKBP15	NA
NA	SPRED1	NA
NA	MAN2B1	NA
NA	MS4A6A	NA
NA	DNASE1L1	NA
NA	TMBIM1	NA
NA	AOAH	NA
NA	ATP6AP2	NA
NA	UBE2D1	NA
NA	TIMP1	NA
NA	SUSD1	NA
NA	VAT1	NA
NA	MYO1E	NA
NA	LACTB	NA

NA	IFIH1	NA
NA	NINJ1	NA
NA	VAMP3	NA
NA	NLN	NA
NA	RBM43	NA
NA	CRTAP	NA
NA	SLC41A2	NA
NA	PRCP	NA
NA	undefined	NA
NA	TMEM63A	NA
NA	GRINA	NA
NA	STAT1	NA
NA	CORO1B	NA
NA	TPMT	NA
NA	CD86	NA
NA	SASH1	NA
NA	VDR	NA
NA	GLIPR2	NA
NA	PLEKHB2	NA
NA	GCH1	NA
NA	TMPPE	NA
NA	MFSD1	NA
NA	MAP3K13	NA
NA	2-Mar	NA
NA	PCK2	NA
NA	RNF13	NA
NA	MLLT4	NA
NA	WDFY3	NA
NA	CLN5	NA
NA	MXD1	NA
NA	CYFIP1	NA
NA	PPP1R15A	NA
NA	LGMN	NA
NA	PLEKHO2	NA
NA	TNFAIP3	NA
NA	ANO6	NA
NA	GALC	NA
NA	ANXA5	NA
NA	SLC35F6	NA
NA	CLIC4	NA
NA	PDLIM5	NA
NA	JAK2	NA
NA	VAC14	NA
NA	ITGB2	NA
NA	CDC42BPB	NA
NA	TSPAN3	NA
NA	IFIT5	NA

NA	RGL1	NA
NA	KLF11	NA
NA	F11R	NA
NA	KDM1B	NA
NA	BACH1	NA
NA	EPSTI1	NA
NA	APOL3	NA
NA	GBP4	NA
NA	GPX1	NA
NA	COLGALT1	NA
NA	ZDHHC7	NA
NA	PLSCR1	NA
NA	GCLC	NA
NA	ITM2B	NA
NA	NAGA	NA
NA	OTUD1	NA
NA	LY6E	NA
NA	SLC43A3	NA
NA	CECR1	NA
NA	GLB1	NA
NA	ANXA4	NA
NA	KIF1B	NA
NA	CPPED1	NA
NA	UHRF1BP1	NA
NA	DUSP5	NA
NA	RIT1	NA
NA	KIAA1598	NA
NA	HPS5	NA
NA	SIDT2	NA
NA	WDFY1	NA
NA	MYD88	NA
NA	PAPSS1	NA
NA	LAPTM5	NA
NA	HLA-DQB1	NA
NA	NUMB	NA
NA	ACOX1	NA
NA	VAMP8	NA
NA	XAF1	NA
NA	ZYX	NA
NA	undefined	NA
NA	CD59	NA
NA	SLC25A40	NA
NA	STX12	NA
NA	DDX60	NA
NA	GSR	NA
NA	ANXA11	NA
NA	LGALS3	NA

NA	CD84	NA
NA	GPCPD1	NA
NA	CREBL2	NA
NA	MPP1	NA
NA	PTTG1IP	NA
NA	CD44	NA
NA	ARAP1	NA
NA	CDS2	NA
NA	PCNX	NA
NA	DOPEY2	NA
NA	CCSAP	NA
NA	TCIRG1	NA
NA	MGAT1	NA
NA	SPG21	NA
NA	ST3GAL1	NA
NA	STAT2	NA
NA	B4GALT1	NA
NA	ZNFX1	NA
NA	NFE2L2	NA
NA	PTPRJ	NA
NA	ARHGAP18	NA
NA	PARP12	NA
NA	ARRB2	NA
NA	SQSTM1	NA
NA	TRIM22	NA
NA	CD97	NA
NA	PRNP	NA
NA	PARP9	NA
NA	CNDP2	NA
NA	TRPV2	NA
NA	TRIM25	NA
NA	NEAT1	NA
NA	PARP14	NA
NA	EIF2AK2	NA
NA	GNPTAB	NA
NA	CD74	NA
NA	ANXA2	NA
